# A cross-cultural re-evaluation of the Exercise Addiction Inventory (EAI) in five countries

**DOI:** 10.1186/s40798-014-0005-5

**Published:** 2015-01-20

**Authors:** Mark D Griffiths, Robert Urbán, Zsolt Demetrovics, Mia B Lichtenstein, Ricardo de la Vega, Bernadette Kun, Roberto Ruiz-Barquín, Jason Youngman, Attila Szabo

**Affiliations:** 1 Nottingham Trent University, Nottingham, UK; 2 Institute of Health Promotion and Sport Sciences, Faculty of Education and Psychology, Eötvös Loránd University, Budapest, Hungary; 3 University of Southern Denmark, Odense, Denmark; 4 Autonomous University of Madrid, Madrid, Spain; 5 University of Miami, Coral Gables, FL USA; 6 Institute of Psychology, Faculty of Education and Psychology, Eötvös Loránd University, Budapest, Hungary

## Abstract

Research into the detrimental effects of excessive exercise has been conceptualized in a number of similar ways, including ‘exercise addiction’, ‘exercise dependence’, ‘obligatory exercising’, ‘exercise abuse’, and ‘compulsive exercise’. Among the most currently used (and psychometrically valid and reliable) instruments is the Exercise Addiction Inventory (EAI). The present study aimed to further explore the psychometric properties of the EAI by combining the datasets of a number of surveys carried out in five different countries (Denmark, Hungary, Spain, UK, and US) that have used the EAI with a total sample size of 6,031 participants. A series of multigroup confirmatory factor analyses (CFAs) were carried out examining configural invariance, metric invariance, and scalar invariance. The CFAs using the combined dataset supported the configural invariance and metric invariance but not scalar invariance. Therefore, EAI factor scores from five countries are not comparable because the use or interpretation of the scale was different in the five nations. However, the covariates of exercise addiction can be studied from a cross-cultural perspective because of the metric invariance of the scale. Gender differences among exercisers in the interpretation of the scale also emerged. The implications of the results are discussed, and it is concluded that the study’s findings will facilitate a more robust and reliable use of the EAI in future research.

## Key points

- Cultural factors prevent the actual (score-based) comparison of Exercise Addiction Inventory (EAI) factor scores.
- The EAI is useful for studying the covariates of exercise addiction in all cultures.
- The interpretation of the EAI (and its items) may be different for men and women.

## Background

Although the beneficial effects of exercise are well known, there is now a growing literature that a small minority of people can experience various negative consequences of excessive exercising [1]. Research into the detrimental effects of excessive exercise has been conceptualized in a number of similar ways, including ‘exercise addiction’ [1], ‘exercise dependence’ [2,3], ‘obligatory exercising’ [4], ‘exercise abuse’ [5], and ‘compulsive exercise’ [6]. To assess the negative effects of excessive exercise, several instruments have been developed and have been extensively reviewed elsewhere [7,8]. Among the most currently used (and psychometrically valid and reliable) instruments are the ‘Obligatory Exercise Questionnaire’ (OEQ) [4,9], the ‘Exercise Dependence Scale’ (EDS) [10,11], the ‘Exercise Dependence Questionnaire’ [12], and the ‘Exercise Addiction Inventory’ (EAI) [13].

The EAI is a short, 6-item instrument aimed at identifying the risk of exercise addiction that has become widely used over the last few years. The EAI assesses six common symptoms of addictive behaviors [14,15] (i.e. salience, mood modification, tolerance, withdrawal symptoms, conflict, and relapse) and has relatively high internal consistency and convergent validity with the Exercise Dependence Questionnaire [13,16,17]. Not only does the EAI have good reliability and validity, but it is theoretically driven, has clear cut-off scores for operationally defining exercise addiction, and is a much shorter scale than other instruments (helping to reduce the time that participants spend completing research surveys).

To date, only one national representative study examining exercise addiction has been carried out [17]. This study surveyed a Hungarian adult population aged 18–64 years (*n* = 2,710), and assessed exercise addiction using both the EAI and the EDS. Results showed that 10.1% (EAI) and 6.2% (EDS) of the population were characterized as non-dependent-symptomatic exercisers, while the proportion of the persons at-risk for exercise dependence was 0.5% (EAI) and 0.3% (EDS). Although there has only been one national study, the EAI has been used to assess exercise addiction in a number of different non-nationally representative subsamples, all of which have confirmed the good psychometric properties of the EAI (see Table 1).

**Table 1:** **Use of the exercise addiction inventory in previously published studies**

**Study**	**Year**	**Sample**	**Measures**	**Prevalence (%) of exercise addiction**
Griffiths et al. [16]	2005	University students	EAI (English)	3.0
Szabo and Griffiths [18]	2007	Habitual exercisers and sport-science students	EAI (English)	3.6 (habitual exercisers); 6.9 (sport-science undergraduates)
Youngman [24]	2007	Triathletes	EAI (English)	19.9
Villella et al. [30]	2010	High-school students	EAI (Italian)	8.5
Lejoyeux et al. [31]	2012	Fitness-centre attendees	EAI (French)	29.6
Mónok et al. [17]	2012	Nationally representative sample (population aged 18–64 years)	EAI (Hungarian)	0.5 (general population); 3.2 (regular exercisers)
Lichtenstein et al. [20]	2013	Fitness exercisers and football players	EAI (Danish)	5.8
Menczel et al. [32]	2013	Fitness-centre attendees	EAI (Hungarian)	1.8 + 1.8 who exhibited both exercise addiction and eating disorders
Szabo et al. [22]	2013	University students and athletes	EAI (Spanish)	7–17

*EAI* Exercise Addiction Inventory.

In a survey of 451 exercisers, Szabo and Griffiths [18] found that 6.9% of British sport-science students (*n* = 261, aged 19–23 years) were at risk of exercise addiction, as measured by the EAI, compared with 3.6% of British gym users (*n* = 194, aged 17–74 years). Warner and Griffiths [19] reported similar results using the EAI. They found that 8% of British gym users (*n* = 100, aged 18–74 years) were exercise addicts. Lichtenstein et al. [20] validated the EAI in Danish by screening 588 exercisers in fitness and football (aged 14–70 years) who exercised for an average of 8 h per week. They reported that the prevalence of exercise addiction in their sample was 5.8%. Another team led by the same author found that exercise addiction prevalence rates in young male footballers (age 18–39 years) were 7.1%, while the figure was higher in general-fitness participants (9.7%) [21].

Using the EAI, Szabo et al. surveyed two Spanish groups of university athletes, including sport students (*n* = 57) and non-sport students (*n* = 90), and a group of ultra-marathon runners (*n* = 95; mean age of the total sample = 27.5 years) [22]. EAI scores were higher in men than women, and ultra-marathoners scored higher on the EAI than both groups of university athletes. The prevalence of being at risk for exercise addictions was 7–10% in university athletes and 17% in ultra-marathoners. They also reported that the amount of exercise was not directly related to exercise addiction. Allegre et al. surveyed 95 French ‘ultra-marathoners’ (who typically run 100-km races) using the EAI and reported only three people (3.2%) as at-risk for exercise addiction [23]. Youngman also investigated the risk for exercise addiction in endurance athletes [24]. The sample comprised 1,285 American male and female triathletes (aged 18–70 years). Approximately 20% of triathletes were classed as being at risk for exercise addiction (with 79% exhibiting some symptoms of exercise addiction). Female triathletes were at greater risk for exercise addiction than male triathletes. As the number of weekly training hours or the number of weekly training sessions increased, so did a triathlete’s risk for exercise addiction.

There is a need to demonstrate the cross-cultural validity of the construct of exercise addiction and its measurement for both theoretical and practical reasons. Testing the cross-cultural properties can highlight the source of differences in cross-national prevalence rates estimated by the EAI. For a meaningful comparison across groups, measurement equivalence or invariance in the constructs underlying one questionnaire across these groups must be demonstrated [25,26]. Different levels of measurement invariance are defined hierarchically, including dimensional, configural, metric, scalar, and strict factorial [25]. Dimensional invariance refers to there being the same numbers of factors present across the comparison groups (e.g. the same number of factors in the EAI across the countries should be present). Configural invariance refers to the same items being related to each factor. In this case, the same items of EAI should define the same factors across the countries. Metric invariance depicts the equivalence of factor loadings that describe the strength of the associations between the specific items and their specific factors. Statistical analysis can reveal if the factor loadings of EAI items are statistically equal, demonstrate that the participants respond to the items in the same way, and that the factors have the same meaning across the countries. Metric invariance is a prerequisite to study factor variance and covariances. Scalar invariance refers to the equivalent intercepts of the items. Scalar invariance is required to compare latent means across groups. The scalar invariance of EAI would be a prerequisite to compare means across groups such as gender, countries, or any other groups. The present study therefore aimed to further explore the psychometric properties of the EAI by combining the datasets of a number of surveys carried out in five different countries (Hungary, UK, Spain, US, and Denmark). This was done to test the assumption that the EAI is invariant across countries and that it can be a useful instrument for future cross-cultural research.

## Methods

### Participants

Data collected in five countries from six previously published studies using the EAI (between 2003 and 2013) were merged to create a new combined dataset (i.e. data from six studies [13,17,18,20,22,24]). These datasets were chosen because all authors, using a non-English version of the EAI worldwide, were contacted for possible collaboration. However, only authors from five nations agreed to share their data and participate in the study. The EAI has also been translated into Italian and French and the authors of these were also invited to collaborate; however, no response was obtained from these researchers therefore no data from those studies were included in the present study. The newly combined dataset provided a sample size of 6,031 participants (2,911 males, 3,095 females, and 25 undisclosed genders). The participant characteristics of each of the six studies are shown in Table 2.

**Table 2:** **Descriptive statistics of exercise addiction inventory data from six samples in five countries, including age, gender, mean exercise addiction inventory score, and individual item analysis**

	**Spain**	**UK**	**US**	**Denmark**	**Hungary**	**Hungary_2**
*N*	266	294	1272	587	583	2,752
Women [*N* (%)]	90 (33.8)	137 (46.6)	684 (53.7)	293 (39.6)	297 (50.7)	1,556 (56.5)
Age [years; mean (SD)]	27.2 (10.61)	25.5 (10.00)	37.9 (9.44)	28.4 (10.74)	29.7 (11.62)	31.5 (8.48)
Exercise addiction score [mean (SD)]	18.6 (4.07)	16.3 (4.45)	20.7 (3.58)	17.6 (3.93)	15.1 (4.72)	17.7 (4.09)
Cronbach’s *α*	0.70	0.80	0.58	0.66	0.73	0.61
Items						
Exercise is the most important thing in my life	3.23 (0.93)	2.30 (1.00)	2.72 (1.03)	2.85 (0.99)	3.00 (1.06)	3.10 (1.02)
Conflicts have arisen between me and my family and/or my partner about the amount of exercise I do	2.23 (1.22)	1.97 (1.10)	2.99 (1.24)	2.09 (1.15)	1.55 (0.97)	1.63 (1.17)
I use exercise as a way of changing my mood	3.75 (1.02)	3.17 (1.11)	3.88 (0.88)	3.42 (1.11)	3.07 (1.31)	3.31 (1.17)
Over time I have increased the amount of exercise I do in a day	3.56 (1.08)	3.18 (1.05)	4.11 (0.81)	3.59 (1.01)	2.52 (1.31)	3.18 (1.31)
If I have to miss an exercise session I feel moody and irritable	2.75 (1.11)	2.52 (1.06)	3.51 (0.99)	2.73 (1.16)	2.19 (1.18)	2.98 (1.24)
If I cut down the amount of exercise I do, and then start again, I always end up exercising as often as I did before	3.03 (1.03)	3.18 (0.99)	3.60 (0.88)	2.90 (1.04)	2.73 (1.34)	3.53 (1.23)

### Measures

Age, gender, and EAI scores were the only measures collated for analysis. The EAI is the shortest psychometrically validated questionnaire to date. It comprises only six statements that correspond to the ‘components’ model of addiction [16]. Each statement is rated on a five-point Likert scale. The statements are coded so that the high scores reflect attributes of addictive exercise behavior: 1 = ‘strongly disagree’, 2 = ‘disagree’, 3 = ‘neither agree nor disagree’, 4 = ‘agree’, 5 = ‘strongly agree’. The six statements that make up the inventory are: (1) “Exercise is the most important thing in my life” (salience); (2) “Conflicts have arisen between me and my family and/or my partner about the amount of exercise I do” (conflict); (3) “I use exercise as a way of changing my mood” (mood modification); (4) “Over time I have increased the amount of exercise I do in a day” (tolerance); (5) “If I have to miss an exercise session I feel moody and irritable” (withdrawal symptoms); and (6) “If I cut down the amount of exercise I do, and then start again, I always end up exercising as often as I did before” (relapse). The EAI cut-off score for individuals considered at-risk of exercise addiction is 24 (out of 30). This cut-off represents those individuals with scores in the top 15% of the total scale score. High scores were considered to be the most problematic for the individual. A score of 13–23 was chosen to be indicative of a potentially symptomatic person, and a score of 0–12 was deemed to indicate an asymptomatic individual.

## Results

### Descriptive statistics

The sample statistics from each of the six studies are presented in Table 3. The samples are varied in sample size, gender distribution, age, and exercise addiction scores.

**Table 3:** **Degree of model fit of the exercise addiction inventory in six samples from five different countries and tests of measurement invariance**

	***χ*** ^**2**^	***df***	***p*** **-Value**	**RMSEA**	**Cfit of RMSEA**	**CFI**	**TLI**	**SRMR**	**Δ** ***χ*** ^**2**^	**Δ** ***df***	***p*** **-Value**	**ΔRMSEA**	**ΔCFI**
Confirmatory factor analysis in each country separately													
Spain	6.1	9	0.727	<0.001	0.946	1.000	1.000	0.022					
UK	32.6	9	<0.001	0.094	0.017	0.942	0.903	0.042					
US	58.4	9	<0.001	0.065	0.051	0.920	0.867	0.033					
Denmark	14.3	9	0.113	0.032	0.828	0.985	0.975	0.024					
Hungary	21.4	9	0.011	0.049	0.491	0.976	0.961	0.027					
Hungary_2	80.5	9	<0.001	0.054	0.266	0.949	0.915	0.027					
Multigroup analyses to test the measurement invariance													
Configural invariance	211.5	54	<0.001	0.055		0.955	0.925	0.029					
Configural vs. metric invariance									114.2	25	<0.001	0.002	0.025
Metric invariance	325.4	79	<0.001	0.057		0.930	0.920	0.051					
Metric vs. scalar invariance									2,140.0	25	<0.001	0.093	0.571
Scalar invariance	2,346.2	104	<0.001	0.150		0.361	0.447	0.136					

The latent variables were identified by fixing one factor loading being equal to 1.

*χ* ^2^ Chi-square value of model fit of each model, *df* degree of freedom, *RMSEA* root mean squared error of approximation, *Cfit of RMSEA* is a statistical test that evaluates the statistical deviation of RMSEA from the value 0.05, and non-significant probability values (*p* > 0.05) indicate acceptable model fit; *CFI* comparative fit index, *TLI* Tucker–Lewis index, *SRMR* the standardized root mean square residual, Δ*χ* ^2^ Satorra–Bentler scaled (S–B scaled) *χ* ^2^ difference test, Δ*df* the difference of df in two models compared, Δ*RMSEA* the difference of RMSEA values in two models compared, Δ*CFI* the difference of CFI values in two models compared.

### Testing measurement invariance across countries

In order to test for measurement invariance across countries, a series of multigroup confirmatory factor analyses (CFAs) with increasing constraints were carried out. To compare the degree of fit of the nested models, the traditional Δ*χ*^2^ test (i.e. the Satorra-Bentler scaled *χ*^2^ difference test) and the recommendations of Cheung and Rensvold [27] and Chen [28] for comparing two nested models were used. Cut-off values of ΔCFI ≤ 0.01 (comparative fit index) and ΔRMSEA ≤ 0.015 (root mean squared error of approximation) were used. The fit indices are reported in Table 3. In order to support dimensional invariance, CFAs of the measurement model in participating countries were separately performed, which resulted in an adequate or acceptable degree of fit. The next step was to test the measurement model freely across all five countries together. This unconstrained solution fitted the data satisfactorily and supported the dimensional and configural invariance. In a further test, the factor loadings were set as equal across countries, and the degree of fit (*χ*^2^) decreased significantly. However, the change in RMSEA was less than the cut-off (≤0.015) but the decrement in CFI was larger than 0.01. The factor loading for each item separately was also tested and, based on ΔRMSEA and ΔCFI values having equal loadings, empirical support for the metric invariance of the scale was found (see Table 4). In the third model, the intercepts were set as equal. The degree of fit (*χ*^2^) decreased significantly again. Furthermore, ΔRMSEA and ΔCFI values were much higher than the cut-off, and therefore scalar invariance of the EAI cannot be claimed. We also tried to identify items that were invariant; hence, we tested each item separately. However, all analyses resulted in higher change scores than the cut-offs (see Table 4), and therefore none of the items demonstrated equal intercepts across the countries.

**Table 4:** **Comparisons of factor loadings and intercepts of the individual Exercise Addiction Inventory items in six samples from five different countries**

**Items**	**Test of equality of factor loadings**				**Test of equality of intercepts**			
	**Δ** ***χ*** ^**2**^	***p*** **-Value**	**ΔRMSEA**	**ΔCFI**	**Δ** ***χ*** ^**2**^	***p*** **-Value**	**ΔRMSEA**	**ΔCFI**
Exercise is the most important thing in my life (item 1)	13.0	<0.03	0.001	0.002	225.9	<0.001	0.027	0.063
Conflicts have arisen between me and my family and/or my partner about the amount of exercise (item 2)	13.7	<0.02	0.001	0.003	1,238.5	<0.001	0.069	0.292
I use exercise as a way of changing my mood (item 3)	34.9	<0.001	0.004	0.007	214.7	<0.001	0.005	0.061
Over time I have increased the amount of exercise I do in a day (item 4)	3.7	0.600	0.001	0.004	179.9	<0.001	0.017	0.247
If I have to miss an exercise session I feel moody and irritable (item 5)	15.7	<0.01	0.001	0.003	153.0	<0.001	0.001	0.042
If I cut down the amount of exercise I do, and then start again, I always end up exercising as often as I did before (item 6)	15.6	<0.01	0.001	0.003	140.8	<0.001	0.004	0.068

For the newly proposed decision criteria, to compare two-nested models the cut-off value of ΔCFI is ≤0.01 and the cut-off value for ΔRMSEA is ≤0.015 [27, 28].

Δ*χ* ^2^ Satorra–Bentler scaled (S–B scaled) *χ* ^2^ difference test, Δ*RMSEA* the difference of root mean squared error of approximation values in two models compared, Δ*CFI* the difference of comparative fit index values in two models compared.

### Testing measurement invariance between men and women

The gender invariance of the EAI in each country was also separately tested. Among the six samples, the change in degree of fit (*χ*^2^) was not significant after constraining factor loadings to be equal in males and females. Furthermore, ΔRMSEA and ΔCFI values were lower than the cut-off values; therefore, metric invariance was supported. However, the scalar invariance was not supported in all countries because degree of fit (*χ*^2^) decreased significantly in all countries, and ΔRMSEA and ΔCFI values were larger than the cut-off scores (see Table 5).

**Table 5:** **Testing gender invariance of the Exercise Addiction Inventory in five different countries: multigroup analyses in six samples**

**Model**		***χ*** ^**2**^	***df***	**RMSEA**	**CFI**	**Δ** ***χ*** ^**2**^	**Δ** ***df***	***p*** **-Value**	**ΔRMSEA**	**ΔCFI**
Spain										
1.	Configural invariance	25.0	18	0.054	0.968					
	Configural vs. metric invariance					7.9	5	0.164	0.003	0.014
2.	Metric invariance	32.9	23	0.057	0.954					
	Metric vs. scalar invariance					20.5	6	<0.003		
3.	Scalar invariance	53.3	29	0.079	0.887				0.022	0.067
UK										
1.	Configural invariance	52.8	18	0.115	0.920					
	Configural vs. metric invariance					7.9	5	0.161	−0.009	0.007
2.	Metric invariance	60.8	23	0.106	0.913					
	Metric vs. scalar invariance					6.7	6	0.353		
3.	Scalar invariance	67.9	29	0.096	0.910				−0.010	0.003
US										
1.	Configural invariance	68.7	16	0.067	0.915					
	Configural vs. metric invariance					8.6	5	0.127	0.006	0.006
2.	Metric invariance	77.0	23	0.061	0.909					
	Metric vs. scalar invariance					51.8	6	<0.001		
3.	Scalar invariance	127.1	29	0.073	0.835				0.012	0.074
Denmark										
1.	Configural invariance	38.7	18	0.063	0.945					
	Configural vs. metric invariance					1.9	5	0.866	0.013	0.011
2.	Metric invariance	39.6	23	0.050	0.956					
	Metric vs. scalar invariance					44.9	6	<0.001	0.030	0.100
3.	Scalar invariance	83.0	29	0.080	0.856					
Hungary										
1.	Configural invariance	29.8	18	0.047	0.977					
	Configural vs. metric invariance					8.6	5	0.128	0.001	0.006
2.	Metric invariance	38.3	23	0.048	0.971					
	Metric vs. scalar invariance					32.8	6	<0.001	0.021	0.049
3.	Scalar invariance	69.8	29	0.069	0.922					
Hungary_2										
1.	Configural invariance	98.8	18	0.057	0.944					
	Configural vs. metric invariance					13.5	5	<0.002	−0.004	0.006
2.	Metric invariance	111.8	23	0.053	0.938					
	Metric vs. scalar invariance					92.1	6	<0.001	0.013	0.057
3.	Scalar invariance	199.6	29	0.065	0.881					

The latent variables were identified by fixing one factor loading being equal to 1.

*df* degree of freedom, *RMSEA* root mean squared error of approximation, *CFI* comparative fit index, Δ*χ* ^*2*^ Satorra–Bentler scaled (S–B scaled) *χ* ^2^difference test, Δ*df* the difference of df in two models compared, Δ*RMSEA* the difference of RMSEA values in two models compared, Δ*CFI* the difference of CFI values in two models compared.

## Discussion

The aim of the present study was to explore the psychometric properties of the EAI by combining six datasets from five different countries (Hungary, UK, Spain, US, and Denmark). The results demonstrated that a one-factor solution was confirmed in data from five countries. The fit indices indicated an excellent degree of fit from data collected in Spain, Denmark, and Hungary, and an adequate level of fit from data collected in the UK and the US. The differences between countries are most likely explained by the undetermined confounding variables due to different sampling methods used by the different research teams. However, testing the factorial structure in the multigroup analysis, the configural invariance was supported; hence, in each country, the one-factor solution is acceptable.

Further analysis of invariance revealed that the liberal criteria for metric invariance supported the equality of factor loadings. Therefore, the covariance analyses are comparable across countries. The scalar invariance is required to compare latent means across groups. This invariance also implies that scales have the same measurement unit and origins; therefore, scores obtained are bias-free and thus can be compared directly [29]. However, analysis of the combined dataset demonstrated that the scalar invariance was not supported across the five countries. This means the intercepts of items were not equal across countries, and that the comparison of EAI scores was biased due to the different use of scales by participants from the five different countries. Put more simply, the EAI factor scores of five countries were not comparable because either the scale was not used in the five countries in the same way, or was not interpreted in the same way by the respondents. However, the covariates of exercise addiction can be studied across countries because the metric invariance was acceptable.

Gender invariance of EAI was also tested in each country separately. Results showed strong support for metric invariance across the gender of exercisers in all samples, but the scalar invariance could not be established. It appears that men and women use starting points of the items (namely intercepts) differently. The consequence of the results obtained is that gender comparison of exercise addiction should be carried out as covariates but that comparison of means directly should be performed cautiously. It can also be considered that different cut-offs should be calculated for men and women.

This is the first study to compare and combine data about exercise addiction (using EAI data) cross-culturally. The study has much strength and is a significant contribution to the exercise addiction literature—particularly in relation to psychometric measurement of the exercise addiction construct. Compared with all previously published studies in the area, the sample size was large and the analysis was both methodical and rigorous. However, there are clearly a number of limitations. The main weakness of the data was that all the data were based on self-report. Such data may be open to recall biases, social desirability biases, and issues surrounding the overall veracity and reliability of the data. The combined dataset and subsequent analyses suggest that the EAI is psychometrically sound but that research teams should be cautious when carrying out cross-cultural research.

## Conclusions

Despite some inter-country differences, the EAI is still an appropriate instrument to assess exercise addiction. However, there is a need to carry out further research on invariance, and the need to consider the development of a cross-culturally invariant measure.
